# A Computational Framework Based on Ensemble Deep Neural Networks for Essential Genes Identification

**DOI:** 10.3390/ijms21239070

**Published:** 2020-11-28

**Authors:** Nguyen Quoc Khanh Le, Duyen Thi Do, Truong Nguyen Khanh Hung, Luu Ho Thanh Lam, Tuan-Tu Huynh, Ngan Thi Kim Nguyen

**Affiliations:** 1 Professional Master Program in Artificial Intelligence in Medicine, College of Medicine, Taipei Medical University, Taipei 106, Taiwan; 2 Research Center for Artificial Intelligence in Medicine, Taipei Medical University, Taipei 106, Taiwan; 3 Translational Imaging Research Center, Taipei Medical University Hospital, Taipei 110, Taiwan; 4 Graduate Institute of Biomedical Informatics, Taipei Medical University, Taipei 106, Taiwan; dothiduyen@tdtu.edu.vn; 5 International Master/Ph.D. Program in Medicine, College of Medicine, Taipei Medical University, Taipei 110, Taiwan; drhung.bvcr@gmail.com (T.N.K.H.); luuhothanhlam2013@gmail.com (L.H.T.L.); 6 Department of Orthopedic and Trauma, Cho Ray Hospital, Ho Chi Minh 70000, Vietnam; 7 Intensive Care Unit, Children’s Hospital 2, Ho Chi Minh 70000, Vietnam; 8 Department of Electrical Engineering, Yuan Ze University, Taoyuan 320, Taiwan; huynhtuantu@saturn.yzu.edu.tw; 9 Department of Electrical Electronic and Mechanical Engineering, Lac Hong University, Dong Nai 76120, Vietnam; 10 School of Nutrition and Health Sciences, Taipei Medical University, Taipei 110, Taiwan; kimngan1702@gmail.com

**Keywords:** essential genetics and genomics, ensemble learning, deep learning, continuous bag of words, DNA sequencing, fastText, prediction model

## Abstract

Essential genes contain key information of genomes that could be the key to a comprehensive understanding of life and evolution. Because of their importance, studies of essential genes have been considered a crucial problem in computational biology. Computational methods for identifying essential genes have become increasingly popular to reduce the cost and time-consumption of traditional experiments. A few models have addressed this problem, but performance is still not satisfactory because of high dimensional features and the use of traditional machine learning algorithms. Thus, there is a need to create a novel model to improve the predictive performance of this problem from DNA sequence features. This study took advantage of a natural language processing (NLP) model in learning biological sequences by treating them as natural language words. To learn the NLP features, a supervised learning model was consequentially employed by an ensemble deep neural network. Our proposed method could identify essential genes with sensitivity, specificity, accuracy, Matthews correlation coefficient (MCC), and area under the receiver operating characteristic curve (AUC) values of 60.2%, 84.6%, 76.3%, 0.449, and 0.814, respectively. The overall performance outperformed the single models without ensemble, as well as the state-of-the-art predictors on the same benchmark dataset. This indicated the effectiveness of the proposed method in determining essential genes, in particular, and other sequencing problems, in general.

## 1. Introduction

Essential genes and their encoded proteins are regarded as the bases of life because they are indispensable for the survival of organisms [1]. Essential genes contain the key information of genomes and therefore could be the key to the comprehensive understanding of life and evolution [2]. Additionally, since essential genes play a critical role in synthetic biology, they are of great importance to genome construction [3]. A comprehensive understanding of essential genes can enable scientists to elucidate the biological nature of microorganisms [4], produce minimal gene subsets [5], develop promising drug targets, and generate potential medicines to combat infectious diseases [6]. Because of their importance, studies of essential genes have been considered crucial in genomics and bioinformatics. Diverse laboratory techniques, e.g., single-gene knockout approaches [7], conditional gene knockouts [8], transposition mutagenesis [9], and RNA interference [10] have been developed to characterize essential genes in microorganisms. Though these experimental methods have many advantages and are relatively reliable, they are still costly and time-consuming.

On the other hand, Rancati et al. [11] recently suggested that the essentiality of genes could change during short- or long-term evolution as a result of changes in environmental and genetic contexts. This could render the prediction of gene essentiality difficult since many factors may affect such a property. The question of how essential genes are indispensable for cells or what underpinning mechanisms regulate their behaviors and functions remains elusive. In addition, with the robust development of experimental techniques, scientists are currently able to access a growing body of experimental data related to genomic sequences, epigenetic patterns, diseases, etc., thus enabling them to conduct more comprehensive research [12]. Therefore, the computational identification of essential genes could shed light on the understanding of its fundamental role in life.

Given the biological meaning of essential genes, a lot of computational models, especially machine learning models, have been developed to identify these genes [13]. Different methods of feature extraction and model construction have been created for this purpose. High throughput genome sequencing and homology mapping offer a diverse group of features for predicting essential genes, including network-based properties [14,15,16], homology [17,18], gene expression [19,20], and functional sites [20]. These features have been used for the construction of several prediction models, including support vector machines (SVMs), decision trees [14], and naïve Bayes classifiers [15]. For example, Deng et al. [21] used hybrid features such as an intrinsic and context-dependent genomic features in an attempt to train an essential gene classification tool. By using ten-fold cross-validation, this model achieved area under the receiver operating characteristic curve (AUC) scores from 0.86 to 0.93 when testing in the same organism and scores from 0.69 to 0.89 for the prediction of cross organisms. In 2005, Chen and Xu successfully combined high throughput data and machine learning approaches to determine protein dispensability in *Saccharomyces cerevisiae* [22]. In 2006, Seringhaus et al. trained a machine learning model using various intrinsic genomic features to detect essential genes in *S. cerevisiae* [23]. Similarly, Yuan et al. developed three machine learning approaches to predict knockout lethality in mice based on informative genomic features [24]. A review of different studies using many computational methods for essential gene prediction in the same organism and other organisms using network features can be seen in the work of Zhang et al. [25]. Lloyd et al. successfully discovered the distinguishing features of the *Arabidopsis thaliana* genome that are helpful for building within- and cross-species predictors, and they subsequently applied this model to detect essential genes in the *Oryza sativa* and *S. cerevisiae* genomes [26]. Additionally, a significant contribution was made by Zhang et al. [27], who combined both sequence- and network-based properties to identify essential genes and found a valuable result. In this research, a deep learning-based model was implemented to learn the features derived from sequence data and protein–protein interaction networks. This research also showed the efficiency of the deep learning model in learning sequence and network-based features. They showed significant improvements in identifying human-essential genes with an average AUC performance of higher than 94%, an area under precision–recall curve of higher than 90%, and an accuracy of higher than 90%.

Though these context-dependent features are linked to gene essentiality, the majority of them mainly depend on experimental omics data, which seem to be unavailable in most microbial species. Additionally, not every feature has a high predictive power, and some features are likely to increase biological redundancy. As a result, most machine learning models based on biological properties tend to be limited to merely a handful of species [28]. For instance, Liu et al. [29] could use biological features to predict essential genes among 31 diverse species, but there were some species that received low accuracies such as *Mycobacterium tuberculosis H37Rv* (19.7%), *Pseudomonas aeruginosa UCBPP-PA14* (18.21%), *Salmonella enterica subsp. enterica serovar Typhimurium str. 14028S* (20.29%), and *Salmonella typhimurium LT2* (27.65%). Essential genes are likely to remain conserved in long-term evolution [30]; therefore, collecting features based on non-biological properties and utilizing them to characterize gene essentiality could help to bridge the gap between organisms with essential genes. In this present study, we addressed the above-mentioned problems in a benchmark dataset in Archaea by extracting intrinsic features from DNA sequence information (biological sub-word features that have been employed from natural language processing (NLP) techniques [31,32,33]) and then subsequently training our ensemble learning-based classifier to make the prediction efficiency better and faster. We also validated the performance results on cross-species datasets to ensure the effectiveness of our model for different species.

## 2. Results and Discussion

### 2.1. Hyperparameters Optimization

**Figure 1:**
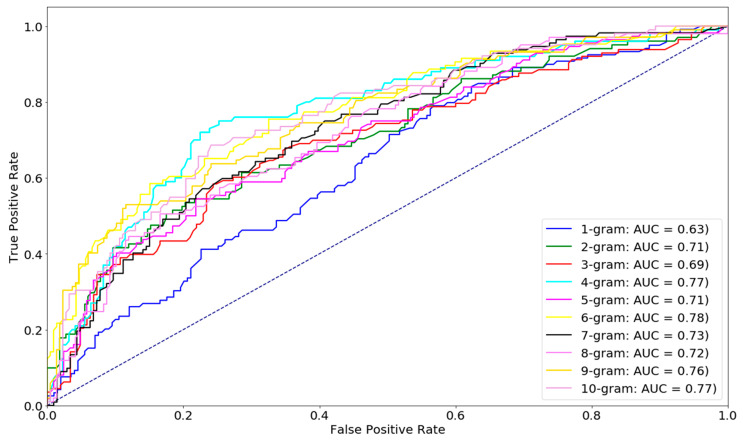
Identification of essential genes at different levels of fastText n-grams. The performance of 6-g (area under the receiver operating characteristic curve (AUC) = 0.78) was better than the other levels.

Hyperparameter optimization plays a very important role in achieving high-quality models in most machine learning and deep learning tasks (e.g., in [31,34]). Since our model was created by combining different algorithms from NLP, machine learning, and deep learning, we also performed pre-experiments on our data to find the optimal parameters of each model. Here, a five-fold cross validation training was also applied to perform this hyperparameter optimization on D1 dataset. First, to deal with fastText, we defined different combined sets from potential hyperparameters that could have reached the best performance. Some tuning parameters were ranged as follows: the number of epochs was from 0 to 500 (step size = 25), the learning rate was from 0.05 to 0.5 (step size = 0.05), wordNgram was from 1 to 10 (step size = 1), and there were three different types of loss functions (ns, hs, and softmax). The other parameters were gathered from previous research [31] on a similar type of fastText applied to DNA sequences (dim = 100, ws = 5). After re-checking all the potential parameters, we found that the most important factor that could affect our model’s performance was the number of sub-word n-grams, as shown in Figure 1. As illustrated in this figure, the performance of the 6 g level slightly outperformed other levels, so we selected 6 g as our optimal n-gram and applied it to further experiments. The other parameters were an epoch of 200, a learning rate of 0.1, and the softmax loss function.

The second optimization step was for different types of machine learning and deep learning techniques. According to the previous experience on k-nearest neighbors (kNN) algorithm, we set k = 10 in our experiments. Furthermore, the “gridsearchCV” was performed in random forest (RF) and SVM classifiers to find the best set for them. In detail, we ranged the number of estimators from 100 to 1000 (step size = 100) and the number of features from 10 to 100 (step size = 10) in RF, while the log2c (for cost) varied between −5 and 5 and log2g (for gamma) varied between −4 and 0 in SVM. For multi-layer perceptron (MLP) and convolutional neural network (CNN) architectures, we performed a comprehensive hyperparameter-tuning process to find their optimal parameters. This process was done by evaluating different combinations of layers and values in a grid search. We report the final parameters in Table 1.

### 2.2. Effectiveness of Ensemble Model

We randomly divided the essential and non-essential gene data into five different sets and performed five-fold cross-validation; Table 2 illustrates the performances of five different predictive modeling approaches using the full 100 features extracted from fastText. The training and testing data here, different from most of the experiments throughout this study, were not exclusive to those prior to exposure. The parameters for each classifier were determined as described in the previous section on hyperparameter optimization. All the five single models—kNN, RF, SVM, MLP, and CNN—demonstrated that the special information of the essential genes could be learned. The CNN and MLP models performed best among the four (with better accuracy, Matthews correlation coefficient (MCC), and AUC values) and was followed closely by the SVM model. Though the superiority was not greater, it still showed that deep neural networks could be a better choice for this kind of dataset because of a sufficient amount of training data and the possibility of generating hidden features of deep neural networks. The measurement metrics strongly showed that we could achieve significant improvement by using deep neural networks for generating the hidden features in comparison with a shallow network.

However, the overall performance of deep neural networks was satisfactory, so an ensemble neural network was applied. This means that during our experiments, we chose these five machine/deep learning methods as basic classifiers and assembled them via a stacking strategy. To verify the ensemble’s effectiveness, we calculated the average accuracies of the individual classifiers by five-fold cross-validation and compared them with the final ensemble model. The final ensemble model was a combination of MLP, CNN, and SVM after we validated the efficiencies by increasing the number of component models (Supplementary Table S1). As presented in Table 2, the ensemble model could achieve a better performance compared to the single models alone. In detail, the ensemble model reached an average sensitivity of 50.2%, a specificity of 90.2%, an accuracy of 77.3%, an MCC of 0.452, and an AUC of 0.814. It could improve by about 2–4% compared to the other single models in terms of all measurement metrics. Therefore, the ensemble model has potential to solve this problem with high performance. It can be used to learn handcraft features better than just a single SVM classifier or a deep learning classifier, as in [31,32].

### 2.3. Comparison with Other State-of-the-Art DNA Sequencing Features

We employed different sequencing features to evaluate the performances of our model in classifying essential genes, including k-mer and different forms of pseudo k-tuple nucleotide composition (PseKNC). PseKNC and k-mer are state-of-the-art feature sets that have been used in a lot of bioinformatics works with high performance, even in previous work on essential genes [35]. Here, we used four common types of PseKNC: PseDNC, PCPseDNC (for dinucleotide), PseTNC (for trinucleotide), and PCPseKNC to extract features from DNA sequences. We inserted these features into our optimal ensemble model and compared the results of each feature type with the performance of our fastText-based features (6 g). Table 3 summarizes the results of the above-mentioned feature extraction techniques and our feature on five-fold cross-validation data. Compared to the other features, our proposed features achieved a higher performance for all the measurement metrics. It is strongly convincing that this NLP feature is efficient at identifying specific problems in essential gene identification.

### 2.4. Imbalance Solving

We aimed to identify essential genes from a set of non-essential genes. Using binary classification, it was important to see that the number of positive samples in each training period was much fewer than the number of negative samples. In other words, our datasets were slightly imbalanced, and this affected the predictive performance. As shown in Table 2, the sensitivity was much lower than specificity, and this suggests that our model could not be considered a good model. It is very important to adjust sensitivity according to imbalance techniques. Therefore, the syntactic minority over-sampling technique (SMOTE) [36] was employed to handle this imbalance. The SMOTE algorithm was implemented by the imblearn package in python, in which we selected SVM as an over-sampling type to generate more data points in positive training datasets. In order to avoid data leakage and overfitting, SMOTE was only applied to a training dataset during the cross-validation procedure. To the end, this process helped our sensitivity come close to the specificity, and the imbalance issue could be solved accordingly. After this step, our model achieved a sensitivity of 60.2%, a specificity of 84.6%, an accuracy of 76.3%, an MCC of 0.449, and an AUC of 0.757. Compared to the original performance, the specificity and AUC were a little bit lower; however, we could boost the sensitivity to create a better model with fewer imbalanced results.

Subsequently, we used the independent dataset to validate the performance of our model on unseen data. A total of 1516 essential genes and 10,499 non-essential genes (D2 dataset) were fed into our final model. Thereafter, our proposed model could predict these sequences with a sensitivity of 79.4%, a specificity of 73.4%, an accuracy of 74.2%, and an MCC of 0.372. Compared to the cross-validation results (in Table 2), the performance was consistent, and it showed that our model did not have an overfitting problem. Another interesting point is that even though we used the original model without the imbalance technique, the independent test results were still balanced between sensitivity and specificity.

### 2.5. Comparison to the Existing Predictors in Identifying Essential Genes

Aiming at evaluating the efficiency of our model in identifying essential genes, we retrieved the results from the other published models that focused on the same problem. For this comparison, the first selected study was the recently reported tool iEsGene-ZCPseKNC [35]. The reason for choosing this study was that it used the same dataset as us (D1), and this made the comparison fair and accurate. iEsGene-ZCPseKNC used Z curve pseudo k-tuple nucleotide composition as their feature and SVM as the machine learning classifier. Furthermore, we also compared our performance results to the other works in terms of methods and features. At testing time, most previous web servers were not publicly available or not available upon request from the authors. Therefore, we decided to replicate their methods on our dataset to make comparisons. There were a variety of sequence-based predictors that were generated using their methods, such as auto covariance, k-mer [37], nucleotide composition [38,39], intrinsic and context-dependent methods [20], information theory [40], and even feature selection on hybrid features [29,41,42]. In summary, the detailed evaluation criteria of both classifiers are presented in Table 4 (including our results of the original and SMOTE versions).

As shown in Table 4, our mode achieved a comparable performance in terms of different measurement metrics compared to the previously published works—especially the work used the same dataset with us (iEsGene-ZCPseKNC). Apart from sensitivity and AUC, our model was improved by at least 4% compared to iEsGene-ZCPseKNC in terms of the other metrics. Moreover, we also validated our performance on the independent test (D2) retrieved from Pheg [39]. This result reached a sensitivity of 79.4%, a specificity of 73.4%, an accuracy of 74.2%, and an MCC of 0.372. Compared to the reported result from Pheg [39], our model achieved more balance than and outperformed the results of Pheg [39] on the same dataset. The results suggested that our ensemble model was more efficient in identifying essential genes than current state-of-the-art models. Therefore, it was observed that the ensemble methods can provide many advantages over these methods such as a reduction in false positive rates and, therefore, better precision. In addition, with the use of biological sub-words, we only needed to use one set of features compared to the others (e.g., the others used a combination of adjacent phase-specific w-nucleotide Z curve variables in the work of Pheg [39] or 1194 features in the work of Zhang et al. [44]). Finally, we also took advantage of an ensemble neural network to boost the predictive performance from the weak learners.

Moreover, because our study only used sequence features, it made it difficult to compare our work with previous work that used different properties such as network- or structure-based ones [27]. To address this issue, we used the dataset from DeepHE [27] that contained sequence-based and network-based features for comparison. There were a total of 16 human essential gene datasets (D3) in this study, and we re-used all of them for analyses. In addition, previous works [27,39] used positive predictive value (PPV) to refine the actual essential genes in their predicted results, so we also took this metric for comparison. As a result, we applied our methods to these 16 datasets and achieved an average PPV of 82.74%. Compared to the results reported in previous works, we reached an improved PPV of 9.69% greater than a sequence-based study [39] and 5% greater than a network-based study [27]. Therefore, the NLP model might hold potential in learning and identifying essential genes from sequence information without the use of network-based features.

Comprehensive comparisons showed a promising result of our ensemble model and NLP-based features in learning and classifying essential genes from sequence information. However, there were also limitations to the comparison that could be addressed and discussed. First, the use of different species datasets made the comparison inaccurate and unfair. However, we highlighted the comparison between our results and the iEsGene-ZCPseKNC’s results because we used the same dataset. Further studies could look at a general dataset that would perform well on different species and make a comparison accurate and fair. Secondly, as mentioned in the literature review, deep learning-based methods that combine both network- and sequence-based properties have been developed [27,45]. Because our study only used sequence features, it could not take full advantage of different properties such as network- or structure-based ones [27]. Therefore, we hope to include more features in further studies to improve our performance results, as well as make accurate comparisons. NLP itself can be combined with deep learning to learn both network- properties and sequence-based properties.

### 2.6. Validation of the Proposed Model on Cross-Species Datasets

**Figure 2:**
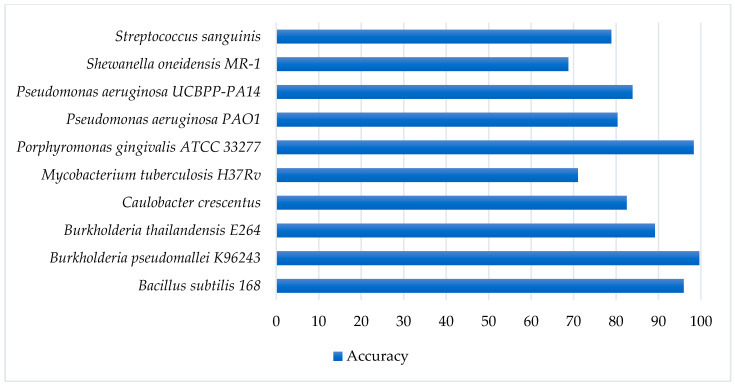
Performance results of identifying essential genes in representative cross-species datasets using the proposed model. Detailed information and predictive accuracy of all species are shown in Supplementary Table S2.

As shown in the study of Liu et al. [29], it was important to evaluate our model on multiple species to generalize our study. Therefore, we also tested the performance of our model on a variety of datasets with different species (D4). In detail, we retrieved the essential genes of 53 diverse bacteria species from DEG [46], which is a large database of essential genes. The validation range was larger than the previous study [29] that had validated on 31 species. Figure 2 shows the predictive accuracies among representative species, and it can be observed that our model performed well on most of the species. There are some species that achieved especially high accuracies such as *Bacillus subtilis 168* (95.9%), *Porphyromonas gingivalis ATCC 33277* (98.27%), *Burkholderia pseudomallei K96243* (99.6%), *Pseudomonas aeruginosa PAO1* (99.4%), and *Agrobacterium fabrum str. C58* (90.3%). Detailed information and predictive accuracy on all species are shown in Supplementary Table S2. We also compared our performance results to a previous study [29] to understand the efficiency of the two models on different species datasets. As shown in Supplementary Table S2, our model was superior to the previous work in terms of the predictive accuracy of essential genes. We also improved the performance for some species that reached a low accuracy in previous work such as *Mycobacterium tuberculosis H37Rv* (71%), *Pseudomonas aeruginosa UCBPP-PA14* (83.88%), *Salmonella enterica subsp. enterica serovar Typhimurium str. 14028S* (53.33%), and *Salmonella typhimurium LT2* (51.3%). Our results also suggested that the hyperparameter optimization learned from gridCV for one species is transferable to other species with acceptable performance. Though there were few datasets that had a weak performance, the large number of high-performance datasets showed the significance of our model in identifying essential genes over multiple species. Future studies could be conducted to create a model to help to improve the predictive performance of all cross-species datasets.

## 3. Materials and Methods

The aims of this study are summarized as follows:

1. A benchmark dataset that was verified and used in previous publication with high confidence was retrieved.
2. The genes were extracted by using an NLP model that aimed to integrate the hidden information of gene sequences.
3. Different machine learning and deep learning-based methods were developed to learn and analyze the extracted features.

**Figure 3:**
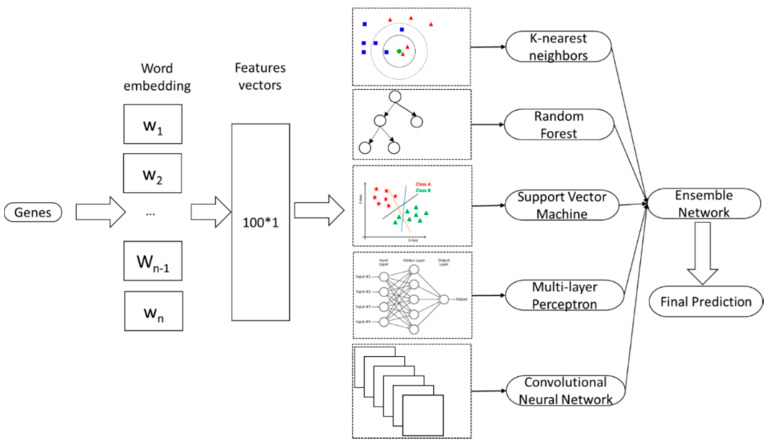
Work flow of the study in identifying essential genes using sequence information. The input was comprised of genes with different lengths and containing different nucleotides. The word-embedding features were extracted by using the fastText package and then learnt by an ensemble deep neural network. After the ensemble network, the output contained binary probabilities to show whether the represented genes belonged to essential genes. Red, green triangles, green circle, blue squares and red pentagons are examples of data points.

The important steps of our proposed method are meticulously described in Figure 3 and the subsequent subsections.

### 3.1. Benchmark Dataset

Generalizing a benchmark dataset is an important part of bioinformatics studies to create an effective prediction model. In this study, we chose the training set defined by Chen et al. [35], which is the most complete and comprehensive dataset of this kind of problem. From this dataset, they retrieved the whole genome of *Methanococcus maripaludis* from the Database of Essential Genes (DEG) (version DEG15.2) [46], which is a comprehensive database containing all the essential genes that are currently available. To minimize the redundancy and eliminate the homology bias that would overestimate the predictor, they excluded the sequences that had >80% similarity in their structures. Finally, the benchmark dataset was represented as:

(1) $$S=S^{+}\cup S^{-}$$

where S is the whole benchmark dataset, $S^{+}$ is the positive subset containing 518 essential genes, $S^{-}$ is the negative subset including 1072 non-essential genes, and the union of these two sets is assigned as ∪. We used this benchmark dataset (namely D1) to train our model on essential gene prediction.

Moreover, we also expanded the validation of the method by retrieving an alternative dataset (namely D2) on human essential gene prediction [39]. The original paper retrieved the dataset from the consensus coding sequence (CCDS) database, and it contained 1516 essential genes and 10,499 non-essential genes. It was used as an independent dataset to evaluate the performance of our model on unseen and cross-species data. Other essential gene datasets were also used in our study, including 16 human datasets (namely D3) and 53 bacteria datasets (namely D4). These D3 and D4 datasets were used to validate the network-based features, as well as the efficiency of diverse species datasets.

### 3.2. Feature Engineering

To effectively extract underlying information from a DNA sequence with minimal features, all the DNA sequences were encoded into biological sub-words features from which the NLP models could be used to capture the hidden information and meaning of biological sequences. As represented, we divided our DNA sequences into different individual nucleotides that could be treated as words in natural sentences. We then trained NLP models to learn and extract information from these biological sequences. Since fastText [47] has recently attracted a lot of attention from biologists [31,48], we also took advantage of this tool in learning our DNA sequences. The features were extracted using the fastText model and the detailed steps are explained as follows:

(1) We split each DNA sequence into a “sentence” comprising biological sub-words (token) in an overlapping manner. A detail explanation of generating different n-gram levels for DNA sequences is shown in Supplementary Figure S1.
(2) We trained the language model to generate word embeddings for each word. No matter the sequence length, the NLP model could generate the word embeddings with the same vector sizes. Thus, we did not apply any padding approach on DNA sequences.
(3) We concatenated these word embeddings to become a vector that represented for whole DNA sequence.
(4) Consequently, these word vectors were fed into our ensemble neural network to evaluate the potential of the network in learning such kinds of features.

### 3.3. Ensemble Neural Network

Our ensemble neural network was the combination of five machine learning and deep learning classifiers, as follows.

#### 3.3.1. k-Nearest Neighbors

kNN is a non-parametric simple algorithm that stores all training samples at the time of learning. In essence, a new case could be characterized based on the number of its neighbors’ votes. Among its k nearest neighbors measured by a distance function, the case is assigned to the most common class. For continuous variables, there are some distance measures—namely Euclidean, Manhattan, and Minkowski. We used Euclidean, the most common distance metric in our experiments. Formula (2) describes the Euclidean distance between *p* and *q*.

(2) $$d\left(p,q\right)=d\left(q,p\right)=\sqrt{{\left(q_{1}-p_{1}\right)}^{2}+{\left(q_{2}-p_{2}\right)}^{2}+\ldots +\;{\left(q_{n}-p_{n}\right)}^{2}}$$

(*p = (p_1_, p_2_... p_n_*) and *q = (q_1_, q_2_... q_n_*) are two points in Euclidean *n*-space)

#### 3.3.2. Random Forest

RF is a classification algorithm based on an ensemble of bagged decision trees with randomly selected features [49]. The RF algorithm embeds the concept of random subset feature selection into bagged decision trees that are trained on random samples with replacement to avoid overfitting. To determine the class of the predicted case, each decision tree is split by a small fraction of randomly selected features and then put through a vote independently under majority rule. In this work, each RF classifier had 500 decision trees. Gini impurity was used to measure the quality of a split by a selected feature, and when looking for the best split, the number of tried features was set to be the square root of the total features.

#### 3.3.3. Support Vector Machine

SVM is a well-known machine learning algorithm that has been used in a variety of fields, especially in bioinformatics. SVM makes linear indivisible samples in original space separable by mapping the original sample features in low-dimensional space to high-dimensional space. It is also a classification method that searches an optimal hyperplane to separate two classes with a maximum margin [50]. In this work, a radial basis function kernel was used for our SVM model, the grid search CV of which was executed to find the optimal kernel parameters for the cost (32768) and gamma (0.00195313).

#### 3.3.4. Multi-Layer Perceptron

MLP, which can distinguish linear non-separable patterns [51], is a feedforward neural network with at least three layers: an input layer, a hidden layer, and an output layer. Compared to a single-layer neural network, MLP adds hidden layers between the input and output layers. For each of our MLP models, there were two fully connected hidden layers with 100 and 50 nodes, while the input nodes represented the biological sub-words vectors and the output layer was used to predict the essential gene outcome.

To simulate how neurons fire, nonlinear activation functions were used. In this work, a rectified linear unit (ReLU) function [52] was used as the activation function in both input and hidden layers because it could overcome the vanishing gradient for non-negative inputs. The ReLU function took the maximum value between 0 and *wTx + b*, where *w* was a weight matrix, *x* was an input, and *b* was a bias term. On the other hand, the output layer for a binary decision adopted a sigmoid function, the outcome of which was strictly increasing between 0 and 1.

Each MLP model is trained to optimize the model parameters (weights) during iteration. Each weight is adjusted based on the error in the next training epoch. To minimize the error measured by cross-entropy loss, the adaptive moment estimation (ADAM) optimization function was applied here during backpropagation. ADAM, which updated model weights using the moving averages of the past gradient and squared gradient, was chosen because it could work effectively for high-dimensional parameter spaces. In this study, 100 training epochs were run with a learning rate of 0.001 without weight decay. Additionally, a 50% dropout rate was applied to each hidden layer to avoid overfitting.

#### 3.3.5. Convolutional Neural Network

A CNN is one of the most commonly used deep learning methods, especially in image recognition. Compared to MLPs, CNNs have additional convolutional and pooling layers. CNNs, which can acquire the spatial relationships of data, can be divided into two sections. The first section extracts features through alternate convolutional filters and pooling layers, and the second section learns through fully connected layers. Many well-known image recognition models like AlexNet, GoogleNet, and ResNet are CNNs. However, it remains unclear how CNNs perform in genomic sequence data. In this study, the CNN models with eight layers were implemented by extending the 1D CNN that was successfully applied in previous work on sequence data [31]. It has been considered as an effective model for learning NLP vector data. In general, CNN models normally comprise an input layer, two convolutional layers, two max-pooling layers, two fully-connected layers, and an output layer.

CNN models can be described simply as follows: the input is a tensor *X f × c*, where *f* represents the number of features and *c* denotes the number of channels. The convolution transforms the input *X* using *m* filter tensors with *q* zero-paddings and *s* strides into *m* feature maps

(3) $$M\in \mathbb{R}\left(\frac{w-n+2q}{s}+1\right)\times \left(\frac{h-n+2q}{S}+1\right)\times m\;\;\;\;\;\;\;\;\;\;$$

where *n* is the order of filter kernels, *q* keeps the same number of channels as the input, and *m* (which determines the number of the output channels) is the number of filter kernels. To effectively reduce the dimensions of feature maps, the pooling layer takes similar steps. Instead of using convolution kernels, the pooling layer transforms the feature map using a pooling filter tensor *P* ∈ ℝ *q* *×* *m* into a pooled feature map, where *q* is the order of pooling kernels and *m* is the channel number of feature maps. Next comes another round of the convolutional and pooling layers. The remaining is the output layer for classification.

To improve performance and mitigate overfitting, an ReLU function [52] as the activation function in the convolutional layers and fully connected layers, a max-pooling filter in the pooling layers, 10% regularization dropout between layers, and a softmax function in the output layer were used. The softmax function is defined as follows:

(4) $$f\left(y=j|x\right)=\frac{exp\left(w_{j}x_{j}\right)}{\sum_{j}exp(w_{j}x_{j})}\;\;\;\;\;\;\;\;\;\;$$

where *x* is the input, *w* is the weight, and *j* is either the essential gene or not. The softmax function normalizes the inputs into a probability space by amplifying the larger ones but suppressing those significantly below the maximum. In our cases, the output layer had two neurons corresponding to predictive possibilities of being genes with essential functions or not.

#### 3.3.6. Ensemble Neural Network

An ensemble neural network could provide other advantages over these methods such as a reduction in false positive rates and, therefore, better precision. Therefore, this study took these advantages to create a stacking ensemble neural network model. This model took the outputs of the base-learners (kNN, RF, SVM, MLP, and CNN) and input them to a metal-learner for second level prediction. At this level, a simple linear regression function was created to predict the outcomes of the input values. Sometimes, due to its smoothing design, the stacking model would outperform each of the individual models, offsetting the shortcomings of the individual models and contributing to the improved predictive results.

Our model was implemented and configured on a machine with i7-9700 CPU (Intel Corporation, CA, USA) and NVIDIA GeForce RTX 2080 Ti GPU (Nvidia Corporation, CA, USA). All machine learning and deep learning were implemented using Python 3, scikit-learn library, and tensorflow. We have freely also released our source codes at https://github.com/khanhlee/eDNN-EG.

### 3.4. Validation Method and Performance Metrics

The experimental design of the rotation of samples between the test and training followed the widely used statistical cross-validation protocol. The purpose of cross-validation is to lower the risk of overestimating or underestimating the true performance of a proposed system, which is achieved by out-of-sample testing. We used cross-validation to train and test the performance of the proposed system. During this process, all the gene sequences were randomly allocated to five subsets with an equal number of samples. Then, we trained five separate recognition systems using four out of the five subsets and performed validation of the fifth hold-out subset. The above-mentioned 5-fold cross-validation procedure was repeatedly conducted 10 times to make our results statistically meaningful, and the average values were then reported as the final results.

The performances of the predictive models were measured by their sensitivity, specificity, accuracy, MCC, and AUC. These evaluation measurements are defined as follows [53]:

(5) $$Sensitivity=\frac{TP}{TP+FN}\;\;\;\;\;\;\;\;\;\;$$

(6) $$Specificity=\frac{TN}{TN+FP}\;\;\;\;\;\;\;\;\;\;$$

(7) $$Accuracy=\frac{TP+TN}{TP+FP+TN+FN}\;\;\;\;\;\;\;\;\;\;$$

(8) $$MCC=\frac{TP*TN-FP*FN}{\sqrt{\left(TP+FP\right)\left(TP+FN\right)\left(TN+FP\right)\left(TN+FN\right)}}\;\;\;\;\;\;\;\;\;\;$$

where *TP*, *TN*, *FP*, and *FN*, respectively, denote true positives, true negatives, false positives, and false negatives. Sensitivity, specificity, and accuracy are presented as the percentage of the correct predictions on different sets of data, i.e., positive data, negative data, and all the data. AUC is used to evaluate the overall performance of a binary classifier. It is based on dynamic positive–negative prediction thresholds instead of a static threshold, as seen in its accuracy. It is the area under receiver operating characteristic (ROC) that plots true positive rates (sensitivity) against false positive rates using different prediction thresholds. The AUC is in the range [0,1], and the higher AUC, the better the classifier. Meanwhile, a perfect classifier has an AUC equal to 1. 

## 4. Conclusions

With advancements in genomic sequence discovery and the abundance of raw sequencing data, there is a requirement for automated tools that can predict the functions of essential genes with high precision and accuracy. In the present research, a machine learning-based approach was built in an attempt to provide comprehensive insights into pathologies, life, and evolution. In detail, we implemented an ensemble neural network to learn the word embedding-based features from genomic sequences and to identify essential genes. Several experiments were conducted, and our optimal model obtained significant results after 10 times of five-fold cross-validation. In detail, our predictor was able to identify essential genes with a sensitivity of 60.2%, a specificity of 84.6%, an accuracy of 76.3%, an MCC of 0.449, and an AUC of 0.814. The proposed performance appeared to be more effective than other feature extractions, as well as previously published works. We also validated our models on a lot of cross-species datasets, and the performance results were significant. In summary, this study suggests that an ensemble neural network and word embedding could comprise a solution for enhancing the performance of essential gene identification. Throughout the research of this topic, biologists and scientists could have more information to study and conduct experiments on essential genes especially.

## Figures and Tables

**Table 1:** Hyperparameters of different machine learning and deep learning classifiers used in this study. KNN: k-nearest neighbors; RF: random forest; SVM: support vector machine; MLP: multi-layer perceptron; and CNN: convolutional neural network.

Classifier	Optimal Parameters
kNN	k = 10
RF	*n*_estimators = 500, *n*_features = 20
SVM	c = 23768, g = 0.001953125
MLP	100–50 nodes, dropout = 0.5, optimizer = adam, learning rate = 0.001
CNN	*n*_filters = 64, dropout = 0.1, optimizer = adadelta

**Table 2:** Five-fold cross-validation performance in identifying essential genes using different machine learning, deep learning, and ensemble learning techniques. MCC: Matthews correlation coefficient.

Classifier	Sens (%)	Spec (%)	Acc (%)	MCC	AUC	Time (s)
kNN	43.5	87.6	73.2	0.348	0.747	0.27
RF	46.9	86.6	73.6	0.367	0.762	12.85
SVM	35.9	92.3	74	0.353	0.775	3.37
MLP	43.5	89.9	74.8	0.385	0.775	94.32
CNN	42.3	90.4	74.7	0.381	0.775	105.18
Ensemble	50.5	90.2	77.3	0.452	0.814	208.12

**Table 3:** Five-fold cross-validation performance in identifying essential genes using different features. PseDNC: pseudo k-tuple dinucleotide composition; PseTNC: pseudo k-tuple trinucleotide composition.

Features	Sens (%)	Spec (%)	Acc (%)	MCC	AUC
k-mer	35.9	90.2	72.4	0.316	0.698
PseDNC	36.5	91	73.2	0.337	0.637
PseTNC	31.7	93.4	73.3	0.331	0.625
PCPseDNC	37.6	89.5	72.6	0.322	0.704
PCPseTNC	33.4	93	73.5	0.341	0.72
fastText	50.5	90.2	77.3	0.452	0.814

**Table 4:** Prediction of essential genes using different state-of-the-art predictors. SMOTE: syntactic minority over-sampling technique; NLP: natural language processing; LASSO: least absolute shrinkage and selection operator; WPCA: weighted principal component analysis.

	Predictors	Feature	Sens	Spec	Acc	MCC
Original	Aromolaran [37]	Auto covariance, pseudo nucleotide composition, k-mer	40.8	90.7	74.4	0.371
	Campos et al. [38]	Nucleotide composition, correlation features	38.5	93	75.2	0.39
	Liu et al. [29]	Sequence-based features and LASSO	41.3	89.7	73.8	0.361
	Tian et al. [41]	Hybrid features	36.5	93.9	75.2	0.389
	Deng et al. [20]	Intrinsic and context-dependent genomic features	34	93.5	74.1	0.355
	Xu et al. [42]	Hybrid features and WPCA	42.7	86.9	72.6	0.331
	Nigatu et al. [40]	Information theoretic features	38.8	92.1	74.8	0.377
	Lin et al. [43]	Hybrid features	35.6	92	73.5	0.345
	Pheg [39]	Nucleotide composition	35	94.4	75.1	0.383
	iEsGene-ZCPseKNC [35]	Nucleotide composition	44.6	89	74.5	0.38
	Ours	NLP-based features	50.5	90.2	77.3	0.452
SMOTE	Aromolaran [37]	Auto covariance, pseudo nucleotide composition, k-mer	55.3	82.2	73.5	0.384
	Campos et al. [38]	Nucleotide composition, correlation features	52.9	85	74.5	0.399
	Liu et al. [29]	Sequence-based features and LASSO	45.2	89.2	74.8	0.389
	Tian et al. [41]	Hybrid features	50.5	85.5	74.1	0.384
	Deng et al. [20]	Intrinsic and context-dependent genomic features	45.2	85.9	72.6	0.341
	Xu et al. [42]	Hybrid features and WPCA	54.8	82.7	73.6	0.386
	Nigatu et al. [40]	Information theoretic features	46.6	86.9	73.8	0.368
	Lin et al. [43]	Hybrid features	44.2	87.8	73.5	0.359
	Pheg [39]	Nucleotide composition	53.8	86.4	75.7	0.426
	iEsGene-ZCPseKNC [35]	Nucleotide composition	63.7	77	72.6	0.396
	Ours	NLP-based features	60.2	84.6	76.3	0.449

## Supplementary Materials

Supplementary materials can be found at https://www.mdpi.com/1422-0067/21/23/9070/s1. Table S1. Comparative performance among different ensemble models; Table S2. Detail information and predictive accuracy of all species datasets; Figure S1. Detail explanation of generating different n-gram levels for DNA sequence (e.g., ‘ATGAC’ and n-gram levels is from 1 to 3).
