# Supplementary material for: A Computational Framework Based on Ensemble Deep Neural Networks for Essential Genes Identification
Source: Int J Mol Sci. 2020 Nov 28;21(23):9070. doi: 10.3390/ijms21239070 (PMC7730808; doi:10.3390/ijms21239070)

Supplementary

A computational framework based on ensemble deep neural networks for essential genes identification

Supplementary Table S1. Comparative performance among different ensemble models

Ensemble model	Sens	Spec	Acc	MCC	AUC
MLP + CNN	42.3	92.5	76.1	0.418	0.811
MLP + CNN + SVM	50.5	90.2	77.3	0.452	0.814
MLP + CNN + SVM + RF	45.6	90.2	75.7	0.409	0.754
MLP + CNN + SVM + RF + kNN	50.5	87.9	75.7	0.417	0.813
CNN + SVM	43.7	90.7	75.4	0.398	0.754
CNN + SVM + RF	39.4	92.5	75.2	0.391	0.797
CNN + SVM + RF + kNN	39.8	93	75.7	0.403	0.756
SVM + RF	36.1	93.5	74.8	0.376	0.774
SVM + RF + kNN	35.9	93.9	75.1	0.384	0.796
RF + kNN	41.3	88.8	73.4	0.347	0.764

Supplementary Table S2. Detail information and predictive accuracy of all species datasets

Species	Essential genes	Our Predicted	Our Accuracy	Previous work
Bacillus subtilis 168	271	260	95.94	47.62
Vibrio cholerae N16961	779	439	56.35	-
Streptococcus pneumoniae	244	154	63.11	53.57
Helicobacter pylori 26695	323	148	45.82	26.89
Mycobacterium tuberculosis H37Rv	614	436	71	19.7
Salmonella typhimurium LT2	230	118	51.30	27.65
Acinetobacter baylyi ADP1	499	125	25.05	58.44
Pseudomonas aeruginosa UCBPP-PA14	335	281	83.88	18.21

Salmonella enterica serovar Typhi	353	211	59.77	55.36
Staphylococcus aureus NCTC 8325	351	56	15.95	62.61
Escherichia coli MG1655 I	609	308	50.57	52.34
Escherichia coli MG1655 II	296	142	47.97	52.34
Caulobacter crescentus	480	396	82.5	61.58
Streptococcus sanguinis	218	172	78.9	52.33
Porphyromonas gingivalis ATCC 33277	463	455	98.27	55.95
Bacteroides thetaiotaomicron VPI-5482	325	197	60.62	35.08
Burkholderia thailandensis E264	406	362	89.16	47.39
Mycobacterium tuberculosis H37Rv II	771	566	73.41	19.7
Salmonella enterica subsp. enterica serovar Typhimurium str. 14028S	105	56	53.33	20.29
Mycobacterium tuberculosis H37Rv III	687	472	68.7	19.7
Sphingomonas wittichii RW1	535	359	67.1	43.55
Shewanella oneidensis MR-1	403	277	68.73	46.75
Pseudomonas aeruginosa PAO1	117	94	80.34	47.39
Salmonella enterica serovar Typhimurium SL1344	353	187	52.97	43.63
Salmonella enterica serovar Typhi Ty2	358	212	59.22	55.36
Bacteroides fragilis 638R	547	320	58.5	30.16
Burkholderia pseudomallei K96243	505	503	99.6	43.17
Pseudomonas aeruginosa PAO1	336	334	99.4	47.39
Streptococcus pyogenes MGAS5448	227	106	46.7	43.15
Streptococcus pyogenes NZ131	241	98	40.66	37.76
Porphyromonas gingivalis ATCC 33277	281	277	98.58	55.95
Synechococcus elongatus PCC 7942	682	606	88.86	-
Rhodopseudomonas palustris CGA009	522	416	79.69	-
Streptococcus agalactiae A909	317	90	28.39	-
Acinetobacter baumannii ATCC 17978	458	150	32.75	-

Acinetobacter baumannii ATCC 17978	156	46	29.49	-
Agrobacterium fabrum str. C58	361	326	90.3	-
Brevundimonas subvibrioides ATCC 15264	412	310	75.24	-
Bacillus thuringiensis BMB171	516	150	29.07	-
Escherichia coli ST131 strain EC958	315	134	42.54	-
Pseudomonas aeruginosa PAO1	551	464	84.21	47.39
Burkholderia cenocepacia K56-2	508	382	75.2	-
Streptococcus mutans UA159	197	61	30.96	-
Neisseria gonorrhoeae MS11	751	475	63.25	-
Escherichia coli O157:H7	1265	428	33.83	-
Ralstonia solanacearum GMI1000	465	383	82.37	-
Streptococcus suis	361	202	55.96	-
Mycobacterium avium subsp. hominissuis strain MAC109	230	157	68.26	-
Providencia stuartii strain BE2467	496	211	42.54	-
Staphylococcus aureus subsp. aureus MSSA476	305	49	16.07	-
Burkholderia cenocepacia J2315	383	235	61.36	-
Vibrio cholerae C6706	343	215	62.68	39.66
Mycoplasma pneumoniae	342	302	88.3	-

'-': dataset was not conducted in the previous work

Supplementary Figure S1. Detail explanation of generating different n-gram levels for DNA sequence (e.g., 'ATGAC' and n-gram levels is from 1 to 3).

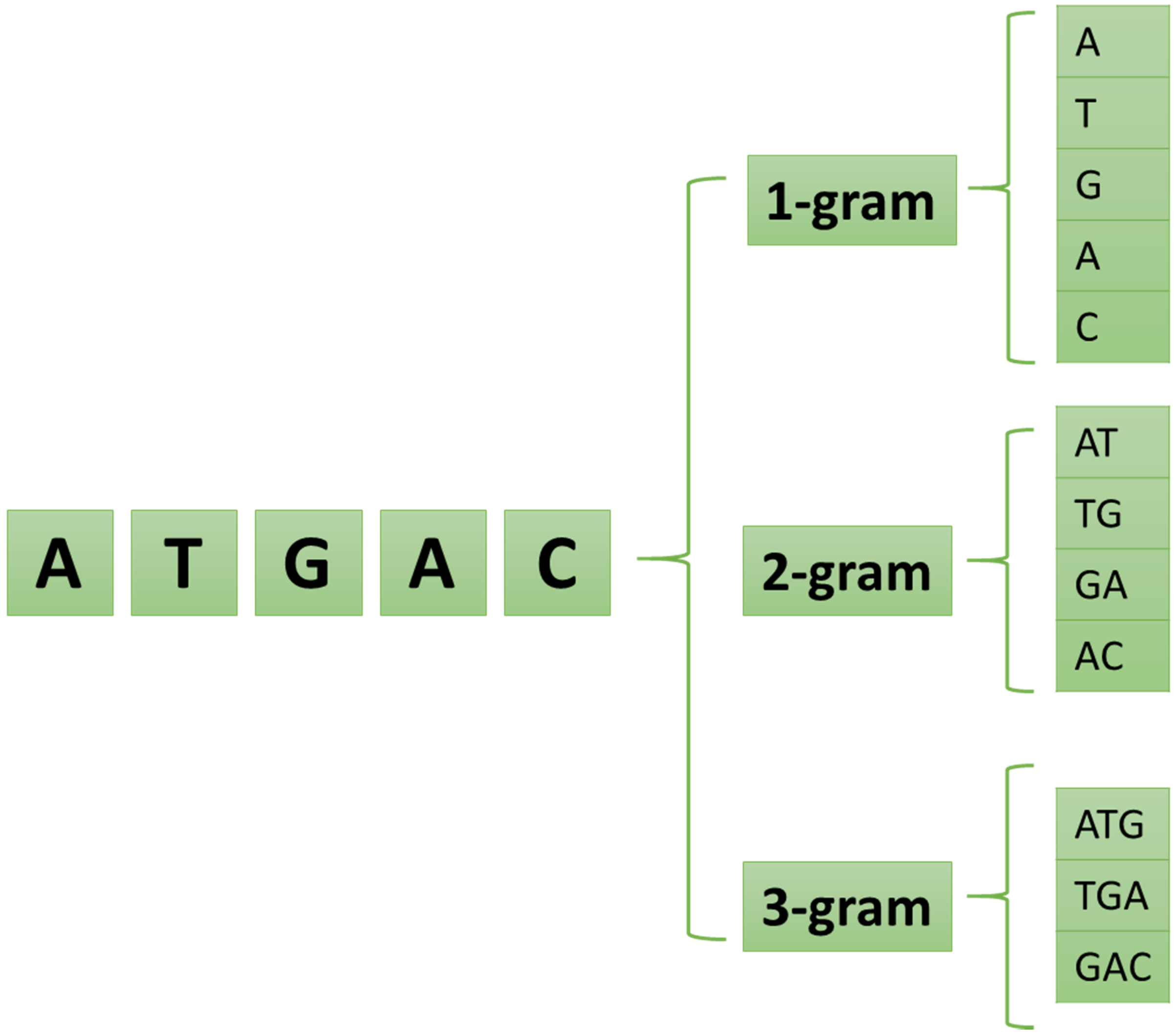

Supplement: Supplementary file 1 [file ijms-21-09070-s001.pdf]
